# *Elsholtzia ciliata* Essential Oil Exhibits a Smooth Muscle Relaxant Effect

**DOI:** 10.3390/ph16101464

**Published:** 2023-10-15

**Authors:** Irma Martišienė, Vilma Zigmantaitė, Lauryna Pudžiuvelytė, Jurga Bernatonienė, Jonas Jurevičius

**Affiliations:** 1 Laboratory of Membrane Biophysics, Institute of Cardiology, Lithuanian University of Health Sciences, Sukilėlių Ave. 15, LT50162 Kaunas, Lithuania; vilma.zigmantaite@lsmuni.lt (V.Z.); jonas.jurevicius@lsmuni.lt (J.J.); 2 Biological Research Center, Lithuanian University of Health Sciences, Tilžės St. 18/7, LT47181 Kaunas, Lithuania; 3 Institute of Pharmaceutical Technologies, Faculty of Pharmacy, Lithuanian University of Health Sciences, Sukilėlių Ave. 13, LT50162 Kaunas, Lithuania; lauryna.pudziuvelyte@lsmuni.lt (L.P.); jurga.bernatoniene@lsmuni.lt (J.B.)

**Keywords:** relaxant effect, *Elsholtzia ciliata* (Thunb.) Hyl., essential oil, herbal medicine, furan derivatives, 2-acetylfuran, 5-methylfurfural, rat prostate, rat aorta

## Abstract

A recent in vivo study in pigs demonstrated the hypotensive properties of essential oil extracted from the blossoming plant *Elsholtzia ciliata*. This study was designed to examine the effect of *E. ciliata* essential oil (EO) on smooth muscle contraction. Tension measurements were performed on prostate strips and intact aortic rings isolated from rats. Results showed that EO caused a concentration-dependent reduction in phenylephrine-induced contraction of both the prostate and aorta, with a more pronounced inhibitory effect in the prostate. The IC_50_ of EO for the prostate was 0.24 ± 0.03 µL/mL (*n* = 10) and for the aorta was 0.72 ± 0.11 µL/mL (*n* = 4, *p* < 0.05 vs. prostate). The chromatographic analysis identified elsholtzia ketone (10.64%) and dehydroelsholtzia ketone (86.23%) as the predominant compounds in the tested EO. Since both compounds feature a furan ring within their molecular structure, other furan ring-containing compounds, 2-acetylfuran (2AF) and 5-methylfurfural (5MFF), were examined. For the first time, our study demonstrated the relaxant effects of 2AF and 5MFF on smooth muscles. Further, results showed that EO, 2AF, and 5MFF altered the responsiveness of prostate smooth muscle cells to phenylephrine. Under control conditions, the EC50 of phenylephrine was 0.18 ± 0.03 µM (*n* = 5), while in the presence of EO, 2AF, or 5MFF, the EC50 values were 0.81 ± 0.3 µM (*n* = 5), 0.89 ± 0.11 µM (*n* = 5), and 0.69 ± 0.23 µM (*n* = 4), respectively, *p* < 0.05 vs. control. Analysis of the affinity of EO for α_1_-adrenergic receptors in the prostate suggested that EO at a certain range of concentrations has a competitive antagonistic effect on α_1_-adrenergic receptors. In conclusion, EO elicits a relaxant effect on smooth muscles which may be related to the inhibition of α_1_-adrenoreceptors.

## 1. Introduction

Traditional and alternative medicine have long relied on the healing properties of herbs and plant-derived compounds to treat various ailments. By exploring herbs and their chemical constituents, researchers constantly seek to discover new compounds that exhibit wide-spectrum therapeutic potential with minimal side effects. One of the most recent areas of our group’s research concerns the investigation of new properties of essential oil extracted from the widespread flowering plant *Elsholtzia ciliata* (Thunb.) Hyl. (EO). Primarily, the antiproliferative activity of EO on three cancer cell lines (human glioblastoma (U87), pancreatic cancer (Panc-1), and triple-negative breast cancer (MDA-MB231)) was determined [1]. Later, studies with isolated rabbit hearts revealed the antiarrhythmic properties typical for first-class antiarrhythmic drugs [2]. Recently, an in vivo study conducted on pigs showed that EO possesses a hypotensive effect, leading to a notable reduction in arterial blood pressure. This decrease in pressure is hypothesized to be a result of EO’s ability to modulate peripheral vascular resistance [3].

Dynamic changes in vascular diameter are primarily influenced by the contractile activation and inactivation processes of contractile proteins within vascular smooth muscle cells [4]. Smooth muscle cells are specialized contractile cells widely distributed throughout the body, including the prostate. Dysfunctions in smooth muscle cells can trigger notable clinical symptoms. For instance, excessive contraction of peripheral vascular smooth muscles can result in hypertension [4]. In the prostate, hyperactivity of these cells is linked to benign prostatic hyperplasia, leading to an enlarged prostate that may obstruct urinary flow [5]. One of the current treatments for these conditions targets the inhibition of smooth muscle contraction via α1-adrenergic receptors [4,5,6]. Therefore, we aimed to examine the effect of EO on rat prostate and aorta and to explore whether the effect is related to α_1_-adrenergic receptors.

**Figure 1:**
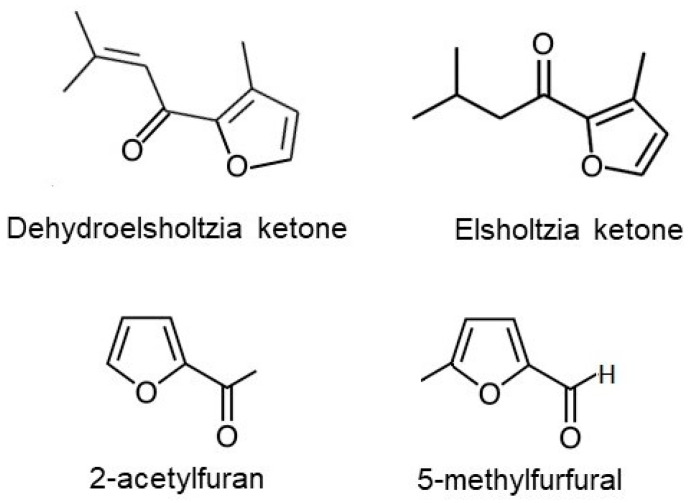
Chemical structures of predominant compounds present in *E. ciliata* essential oil, dehydroelsholtzia ketone and elsholtzia ketone, and furan derivatives, 2-acetylfuran and 5-methylfurfural.

Earlier research showed that among the 48 organic compounds identified in EO extracted from plants collected in Lithuania, 92% were ketones. The most prevalent of these were dehydroelsholtzia ketone and elsholtzia ketone [1]. From a structural viewpoint, both of these compounds are classified as oxygenated monoterpenes [7]. However, both compounds also feature a furan ring within their molecular structure. A diverse range of furan ring-containing compounds can be found in food, including herbs. These structures are also prominent in both synthetic and natural medicinal products. It is worth noting that many furan-containing compounds are known to be toxic [8]. However, toxicity is not universal to all furan derivatives. A lot of natural furan derivatives are declared to have antimicrobial and anti-inflammatory effects [9]. Some furan-containing compounds have therapeutic applications in systematic diseases. For example, prazosin, an α_1_-adrenergic receptor antagonist, medication prescribed for the treatment of several diseases including hypertension and benign prostatic hyperplasia [10], contains a furan ring. Furosemide, an inhibitor of Na^+^-K^+^-2Cl^−^ cotransporter-2, a potent diuretic agent applied to decrease extracellular fluid volume expansion in heart and kidney disease [11], also features this ring in its molecular design. It encouraged us to investigate the effects of other furan-containing compounds on rat prostate and aorta smooth muscles. We aimed to compare these effects with those of EO and determine whether the effects of EO are influenced by its dominant ketones. For this purpose, we chose 2-acetylfuran (2-furyl methyl ketone, 2AF) and 5-methylfurfural (5-methyl-2-furaldehyde, 5MFF). These compounds share structural similarities with the predominant ketones found in EO (Figure 1). They are also known as flavoring substances [12] and are readily available.

Our paper presents research data on tension measurements of rat prostate and aorta revealing the relaxant effect of EO on smooth muscles. This study for the first time demonstrates the relaxant effect of furan ring-containing compounds, 2AF and 5MFF, on smooth muscles of both the aorta and the prostate. Analysis of the affinity of EO for α_1_-adrenergic receptors in the prostate suggests that observed effects may be related to inhibitory action on α1-adrenergic receptors. This physiologically relevant effect, together with the previously established antiarrhythmic and hypotensive effects contributes to research on the potential application of EO in the treatment of various pathologies.

## 2. Results

### 2.1. The Qualitative and Quantitative Composition of EO

**Figure 2:**
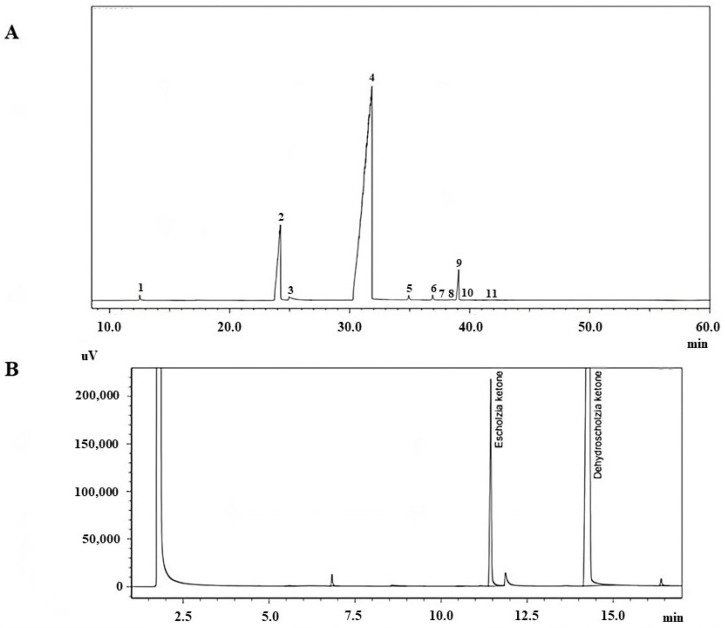
*E. ciliata* essential oil chromatograms obtained by gas chromatography–mass spectrometry (**A**) and gas chromatography with flame ionization detection (**B**). 1—cineol (RI = 1030), 2—elsholtzia ketone (RI = 1201), 3—alfa-dehydroelsholtzione (RI = 1217), 4—dehydroelsholtzia ketone (RI = 1301), 5—beta-bourbonene (RI = 1378), 6—beta-caryophyllene (RI = 1381), 7—8-isopropyl-1methyl-5-methylene (RI = 1383), 8—isogermacrene D (RI = 1385), 9—alfa-humulene (RI = 1446), 10—germacrene D (RI = 1480), 11—bicyclogermacrene (RI = 1493); RI—retention index.

The chemical composition of herbal extracts can be influenced by environmental factors, differences in growing locations, and seasons. Therefore, we first analyzed the composition of the EO used in this study by using chromatographic analysis methods, i.e., gas chromatography–mass spectrometry (GC–MS) and gas chromatography with flame ionization detection (GC-FID). According to our analysis, the main compounds of EO were sesquiterpenes and ketones. Elsholtzia ketone (10.64%) and dehydroelsholtzia ketone (86.23%) were the predominant compounds in the essential oil composition. Cineol, beta-bourbonene, beta-caryophyllene, and alfa-humulene were also identified in essential oil (Figure 2A). GC-FID analysis revealed that the predominant compounds of EO are elsholtzia and dehydroelsholtzia ketones (Figure 2B). The Elsholtzia ketone amount in EO was 1221.49 µg/mL (according to eugenol) and dehydroelsholtzia ketone was 7409.97 µg/mL (according to eugenol). These results are similar to our previous analysis [1].

### 2.2. Effect of EO on Prostate and Aorta Contraction

**Figure 3:**
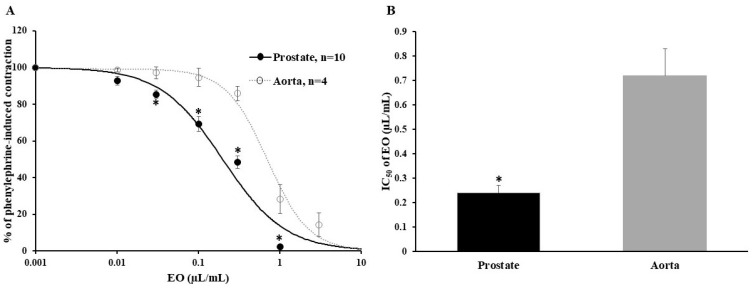
Inhibition of phenylephrine-induced contraction by increasing concentrations of *E. ciliata* essential oil (EO) (**A**) and the half-maximal inhibitory concentrations (IC_50_) of EO on phenylephrine-induced contraction (**B**) in the rat prostate and aorta. Symbols, columns, and bars represent mean ± SEM, * *p* < 0.05 vs. aorta, n indicates the number of prostate strips or aorta rings. Lines indicate the data fitted to the Hill equation.

EO caused a concentration-dependent reduction in the phenylephrine-induced contraction of the prostate strips and intact aortic rings. In the prostate, a significant relaxation effect was observed starting from a concentration of 0.03 µL/mL, while in the aorta the tension significantly decreased from 0.3 µL/mL of EO (Figure 3A). The IC_50_ of EO for the prostate was 0.24 ± 0.03 µL/mL (*n* = 10), and for the aorta it was 0.72 ± 0.11 µL/mL (*n* = 4, *p* < 0.05 vs. prostate) (Figure 3B). The data showed that the inhibitory effect of EO on phenylephrine-induced contraction was significantly more pronounced in the prostate compared to the aorta.

### 2.3. Effect of 2AF and 5MFF on Prostate and Aorta Contraction

**Figure 4:**
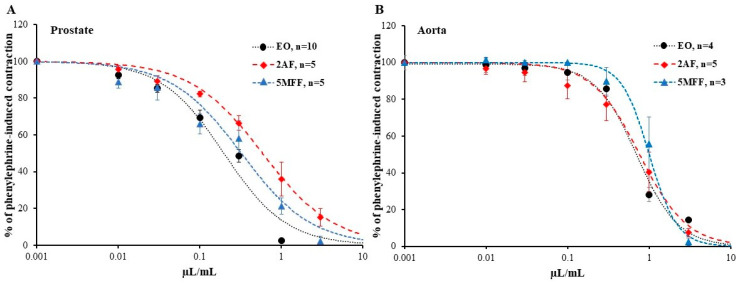
Inhibition of phenylephrine-induced prostate (**A**) and aorta (**B**) contraction by increasing concentrations of *E. ciliata* essential oil (EO), 2-acetylfuran (2AF), and 5-methylfurfural (5MFF). Symbols and bars represent mean ± SEM; n indicates the number of prostate strips or aorta rings. Lines indicate the data fitted to the Hill equation.

The effects of 2AF and 5MFF on the contraction of prostate strips and aortic rings were examined. Like EO, both 2AF and 5MFF caused a concentration-dependent reduction in the phenylephrine-induced contraction of both the prostate and aorta (Figure 4). The contraction of the prostate significantly began to decline starting from a concentration of 0.03 µL/mL of 2AF and 5MFF, while the contraction of the aorta did so from 0.3 µL/mL of 2AF and 1 µL/mL of 5MFF. The IC_50_ of all tested compounds on phenylephrine-induced contraction in both the prostate and aorta are presented in Table 1. The data showed that 2AF and 5MFF caused half of the maximal relaxation of the precontracted prostate at a higher concentration compared to EO. However, the effect of 2AF and 5MFF on aorta contraction was similar to that of EO, i.e., all the compounds induced half of the maximal relaxation of the precontracted aorta at similar concentrations. Comparing the inhibitory effect of tested compounds in the prostate and aorta, the results showed that smooth muscles in the prostate were more sensitive to the effects of 5MFF than in the aorta, while the effects of 2AF were not significantly different in both tissues.

### 2.4. Effect of EO, 2AF, and 5MFF on Dose-Response Curves for Phenylephrine in the Prostate 

**Figure 5:**
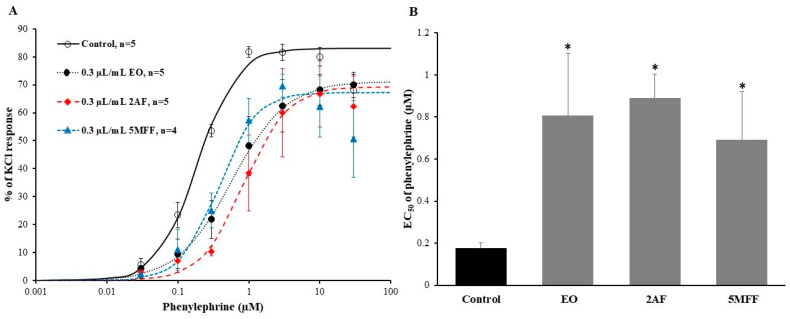
Dose-response curves for phenylephrine (**A**) and the half-maximal effective concentrations (EC_50_) of phenylephrine (**B**) in the rat prostate in the absence (control) and presence of 0.3 µL/mL of *E. ciliata* essential oil (EO) or 2-acetylfuran (2AF), or 5-methylfurfural (5MFF). Symbols, columns, and bars represent mean ± SEM, * *p* < 0.05 vs. control; n indicates the number of prostate strips. Lines indicate the data fitted to the Hill equation.

The prostate smooth muscle cells, responsible for organ contractility, express high levels of α_1_-adrenergic receptors [13]. The observed relaxing effect of EO and other structurally related compounds on phenylephrine-induced contraction suggested that these compounds may affect α_1_-adrenergic receptors. To investigate this, a series of experiments were conducted in the rat prostate to assess the dose-response of phenylephrine under control conditions and in the presence of 0.3 µL/mL EO, 2AF, or 5MFF. Results showed that EO, 2AF, and 5MFF caused a rightward shift in the dose-response curves for phenylephrine (Figure 5A). Under control conditions, the maximal phenylephrine-induced contraction force was 78.58 ± 2.82% of KCl response (*n* = 5). E_max_ caused by phenylephrine in the presence of EO, 2AF, or 5MFF was 74.13 ± 6.05% (*n* = 5), 73.46 ± 6.12% (*n* = 5), and 71.93 ± 6.43% (*n* = 4) of KCl response, respectively, and was not significantly different from the control. However, in the presence of the tested compound, the concentrations of phenylephrine required to produce half of its maximum effect (EC50) were significantly higher. Under control conditions, the EC_50_ value was 0.18 ± 0.03 µM (*n* = 5), while in the presence of EO, 2AF, or 5MFF, the EC50 values were 0.81 ± 0.3 µM (*n* = 5), 0.89 ± 0.11 µM (*n* = 5), and 0.69 ± 0.23 µM (*n* = 4), respectively, *p* < 0.05 vs. control (Figure 5B).

Results show that tested compounds altered the responsiveness of smooth muscle cells in the prostate to phenylephrine. A significant rightward shift of dose-response curves for phenylephrine, which is a selective agonist of α_1_-adrenergic receptors, may indicate a competitive inhibition of these receptors by EO, 2AF, and 5MFF.

### 2.5. Antagonistic Effects of EO on Phenylephrine-Induced Contractions in the Prostate

To estimate the affinity of EO for α_1_-adrenergic receptors in the prostate, the dose-response curves were obtained for phenylephrine in the presence of 0.1, 0.3, and 1 µL/mL of EO. EO shifted the phenylephrine concentration-effect curves to the right. EC_50_ of phenylephrine at 0.1 and 1 µL/mL of EO was 0.42 ± 0.06 µM (*n* = 4) and 2.89 ± 0.5 µM (*n* = 3), *p* < 0.05 vs. control, respectively. E_max_ at 0.1 µL/mL of EO was 74.09 ± 11.4% (*n* = 4) and did not significantly differ from the control, while at 1 µL/mL it was significantly reduced to 48.11 ± 12.2% of KCl response (*n* = 3). EC_50_ and E_max_ values obtained in the presence of 0.3 µL/mL of EO were presented above.

**Figure 6:**
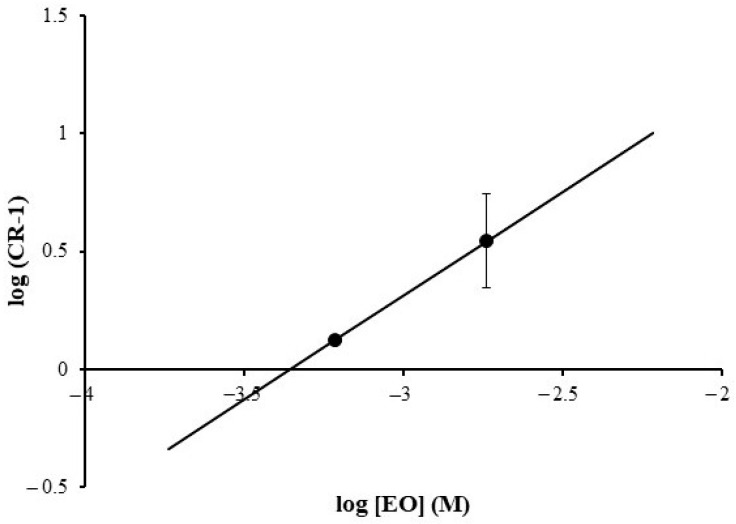
Schild plot analysis for *E. ciliata* essential oil (EO) against the effect of phenylephrine in the rat prostate. EO concentration was calculated using the molecular weight of the dominant dehydroelsholtzia ketone in EO. CR represents the EC_50_ ratio which was calculated by dividing EC_50_ of phenylephrine obtained after EO treatment by EC_50_ obtained under control.

The averaged data of phenylephrine concentration effect at the presence of concentrations of EO which did not affect the maximal effect of phenylephrine, i.e., 0.1 and 0.3 µL/mL, were used in the Schild regression analysis. The molecular weight of the dominant dehydroelsholtzia ketone in EO was taken into account when creating the plot. Analysis revealed that the slope was 0.88 and the pA_2_ value was 3.36 (Figure 6). The data suggest that EO at a certain range of concentrations has a competitive antagonistic effect on α_1_-adrenergic receptors in the prostate.

## 3. Discussion

The primary objective of our study was to examine the effect of EO on smooth muscle contraction. Experiments were carried out on the intact thoracic aorta and prostate ventral lobe of the rat. The concentration-dependent relaxant effect was obtained. This finding potentially elucidates the marked reduction in blood pressure witnessed post-EO injection in pigs during in vivo studies [3]. Interestingly, our study revealed a different impact of EO on tested muscle tissues. The reduction in phenylephrine-induced contraction by EO was observed to be more prominent in the prostate as compared to the aorta. This distinction may be associated with the α_1_-adrenoceptors. These receptors, predominantly found in vascular smooth muscles, play a crucial role in local vasoconstriction which, in turn, affects the regulation of both blood pressure and temperature. Moreover, they influence the contraction behavior of the prostate and bladder neck [14]. It has been determined that contractile properties of the prostate are predominantly mediated via the α_1A_-adrenoreceptors subtype [15]. Conversely, in the aorta, α_1D_-adrenoreceptors dominate over other subtypes [14,16,17]. This variance is of great importance to researchers, especially when characterizing the antagonistic properties of certain drugs. The prostate and aorta are used as typical samples for α_1A_- and α_1D_- subtypes of α_1_-adrenoreceptors, respectively [18]. A noteworthy point of consideration in our study design was the selection of aged rats as subjects. Earlier studies have shown that with aging, there is an increase in the expression levels of α_1A_-adrenergic receptors, along with α_1D_-adrenergic receptors, in the human prostate [15]. This increase is often concomitant with benign prostatic hyperplasia [19,20]. Given this background, the evidence from our study suggests that the EO relaxing effect could likely be attributed to its antagonistic impact on the α_1_-adrenergic receptors, most probably on the α_1A_ subtype.

*E. ciliata*, also known as Vietnamese mint, is a plant that contains a variety of active compounds. These compounds impart its unique aroma and potential medicinal properties. These active compounds are a major reason why this plant is widely used in both culinary cuisine and traditional medicine. Essential oils are typically rich in terpenoids: monoterpenes, sesquiterpenes, and diterpenes [21]. Our analysis confirmed previously reported data, showing that the predominant compounds in EO are elsholtzia and dehydroelsholtzia ketones. It was hypothesized that the effects of EO may be determined by its dominant ketones, having a furan ring in their molecular structure. Following this hypothesis, other furan ring containing compounds, 2AF and 5MFF, were examined. Furans are a class of heterocyclic aromatic chemicals characterized by a pentagonal ring structure consisting of one oxygen atom and four carbon atoms. Furan itself is a colorless, volatile liquid with slight toxicity. Furan-based pharmaceuticals offer a diverse range of potential therapeutic benefits, including antibacterial, anti-inflammatory, anti-tumor, analgesic, muscle relaxant, antihypertensive, diuretic, etc. [8,9]. Earlier studies demonstrated that EO possesses features of class 1B antiarrhythmic drugs [2]. However, there are no data about the physiological effects of 2AF and 5MFF. Both 2AF and 5MFF are widely used as flavoring agents, and 2AF is also used as an intermediate in the synthesis process of certain antibiotics [12]. Our study for the first time demonstrated the relaxant effect of these widely used compounds on smooth muscles of both the aorta and the prostate. Compared to EO, the effect of 2AF and 5MFF on the aorta was similar but milder on the prostate. Such findings suggest that the effect of EO may be associated with the furan ring. However, the longer chain in dehydroelsholtzia ketone and elsholtzia ketone or the presence of other active constituents in the essential oil may determine a more potent and targeted effect on the prostate.

The more pronounced impact of EO, 2AF, and 5MFF on the smooth muscle of the prostate prompted us to examine the effect of these compounds on concentration-response curves for phenylephrine, a selective α_1_-adrenoreceptor agonist, in the prostate. Results showed typical characteristics of antagonists including right shifts of concentration-response curves, increased EC_50_ values for phenylephrine, and no significant change in E_max_. The results supported the hypothesis that the relaxing effect of EO, 2AF, and 5MFF is related to their inhibitory effect on the α_1_-adrenoreceptors. Further investigation of the EO antagonistic effect revealed that only at lower concentrations (0.1, 0.3 µL/mL) EO acts as a concurrent inhibitor of α_1_-adrenoreceptors since at higher concentrations (1 µL/mL) it significantly influences not only EC_50_ but also E_max_. The interaction points for α1-adrenoreceptors antagonists have been found to vary depending on the molecular structure of the antagonist. Unlike agonist binding, which takes place near the core interior of the receptor, known selective α1-adrenoreceptors antagonists, like the furan ring-containing prazosin, interact nearer the receptor’s extracellular surface [22,23]. Given the lipophilic nature of EO and other examined furan derivatives, it is likely that they can affect the affinity for agonists by directly interacting with certain amino acid residues that are exposed to the phospholipid layer of the membrane, or indirectly by inducing receptor structural modifications due to their incorporation into the membrane. There is also the possibility that these compounds may enter the cell cytoplasm and target intracellular components involved in the relaxation process of smooth muscles. Previous electrophysiological studies with Langendorff-perfused rabbit hearts revealed that EO at a higher concentration (0.3 µL/mL) shortened action potentials and slowed the impulse propagation down via the atrioventricular node, inducing an conduction block. These results suggested that EO can have an inhibitory effect not only on Na^+^-current but also the Ca^2+^-current [2]. Another study found that the furan derivative, 5-hydroxymethylfurfural, relaxed coronary arteries in a concentration-dependent manner and exerted negative inotropic, lusitropic, and chronotropic effects in isolated rat hearts. The authors concluded that these effects were mediated by the inhibition of L-type Ca^2+^ channels [24]. Experiments conducted on *Xenopus* oocytes with expressed human aquaporin-1 showed that 5-hydroxymethylfurfural and other structurally related, furan ring-containing, compounds tested blocked the ion conductance through these channels [25]. Collectively, previous findings support the assumption that EO, 2AF, and 5MFF might be interacting with multiple targets present within the smooth muscle cells.

α_1_-adrenoreceptor antagonists play a pivotal role in modulating intracellular calcium flow, subsequently leading to the relaxation of smooth muscles. Their efficacy in this area has made them therapeutic agents for the treatment of benign prostatic hyperplasia [26,27], as well as hypertension [4]. Given the prevalence of benign prostatic hyperplasia among senior men who often have other comorbidities including hypertension, the pharmaceutical world is increasingly intrigued by drugs with multiple targets. Great attention is paid to plant-based pharmaceuticals due to the belief that naturally sourced medications might have a gentler side effect profile. Our study demonstrates that EO has a potent relaxing influence on smooth muscles in the prostate and aorta which may be related to the inhibition of α_1_-adrenoreceptors. This physiologically relevant effect, together with the previously identified antiarrhythmic and hypotensive effects [1,2], sketches a promising picture of EO’s potential therapeutic applications across a diverse spectrum of medical conditions. 

## 4. Materials and Methods

### 4.1. Animals

Male Wistar rats weighing 500–700 g (20–24 weeks old) were used in the study. Animals were housed in a controlled environment with suitable conditions, i.e., relative air humidity, temperature, and a fixed 12-h light-dark cycle, and they had food and water ad libitum. The study was conducted in accordance with the guidelines outlined in the EU Directive 2010/63/EU [28] for the protection of animals used for scientific purposes and was approved by the Lithuanian Commission on the Ethics of the Use of Experimental Animals at the State Food and Veterinary Services under Permission No. G2-199. 

### 4.2. Materials and Chemicals

Essential oil of *E. ciliata* (EO) was prepared from the dried herb, collected in Vilnius, Lithuania, in August 2022, at the Lithuanian University of Health Sciences, as reported previously [1]. The qualitative and quantitative composition of *E. ciliata* essential oil was evaluated by chromatographic analysis methods. Qualitative gas chromatography–mass spectrometry (GC–MS) analyses were performed by using the gas chromatograph-mass spectrometer system Shimadzu GC–MSQP2010 (Shimadzu, Tokyo, Japan) equipped with a Shimadzu autoinjector AOC-5000 (Shimadzu, Tokyo, Japan) according to Pudziuvelyte et. al. method [29]. The capillary column for analysis used was RXI-5MS (30 m × 0.25 mm i.d. × 0.25 μm film thickness). The operational conditions included a temperature program from 50 °C (5 min) to 200 °C at 2 °C/min and to 315 °C (15 min) at 15 °C/min. The gas chromatograph-mass spectrometer was equipped with a split/splitless injector (260 °C), and the split ratio was 1:60, with inlet pressure 69.4 kPa. The carrier gas was helium (purity > 99%), delivered at a constant linear velocity of 40 cm/s. The interface temperature was 280 °C, and the MS ionization mode was electron ionization. The detector voltage was 0.99 kV, the acquisition mass range was 29–500 u, the scan speed was 2500 amu/s, and the acquisition mode was full scan with scan interval 0.20 s.

Gas chromatography with flame ionization detection (GC-FID) analyses were performed by using the gas chromatograph Shimadzu GC-2010 (Shimadzu, Tokyo, Japan) equipped with a Shimadzu autoinjector AOC-20is (Shimadzu, Tokyo, Japan) according to Pudziuvelyte et al.’s [29] method. The operational conditions were as follows: temperature increased from 70 °C (3 min) to 180 °C (5 min) at 5 °C/min, then increased to 250 °C (3 min) at 10 °C/min and to 315 °C (10 min) at 10 °C/min. A capillary column RXI-5MS (30 m × 0.25 mm i.d. × 0.25 μm film thickness) was used. The split injector temperature was 260 °C, split ratio was 1:20, and inlet pressure was 104.0 kPa. The carrier gas was helium (purity > 99%), delivered at constant linear velocity of 30.1 cm/s. FID (320 °C) gases were helium (flow 40.0 mL/min), air (flow 400.0 mL/min), and helium (as make up, flow 30.0 mL/min). Quantitative analysis was carried out by using the external standard method. Elsholtzia (C_10_H_14_O_2_, MW 162 g/mol) and dehydroelsholtzia (C_10_H_12_O_2_, MW 164 g/mol) ketone contents were calculated using the equation of linear calibration of eugenol (C_10_H_12_O_2_, MW 164 g/mol). The linear calibration curve was constructed as an area vs. concentration (eugenol R^2^ = 0.9999).

The 2-acetylfuran (99%, 2AF), 5-methylfurfural (99%, 5MFF), and all other chemicals were purchased from Sigma-Aldrich (Schnelldorf, Germany). Prior to every experiment, fresh stock solutions of EO, 2AF, and 5MFF were prepared by diluting them with ethanol (anhydrous, ≥99.8%) in a 1:1 ratio. The highest applied concentration of EO, 2AF, and 5MFF was 3 µL/mL, with a solvent concentration of 0.3%.

### 4.3. Preparation of Aortic Rings and Prostate Strips

Rats were anesthetized via intraperitoneal injection of ketamine hydrochloride (90 mg/kg, Ketamidor, Richter Pharma, Wels, Austria) and xylazine hydrochloride (9 mg/kg, Sedaxylan, Eurovet, Lublin, Poland). After anesthesia animals were killed by applying cervical dislocation, and the descending thoracic aorta and prostate were dissected. The organs were immediately immersed in a cooled (~2 °C) oxygenated (100% O_2_) Tyrode’s solution (in mM: 135 NaCl, 5.4 KCl, 1.8 CaCl_2_, 0.9 MgCl_2_, 0.33 NaH_2_PO_4_, 10 glucose, and 10 HEPES, pH 7.4). The aorta was separated from surrounding connective tissue and fat and then cut into 2–2.5 mm rings. The ventral lobe of the prostate was cut into strips (1.5–2 mm in width, 2–3 mm in length), and the ends of muscle strips were tied with cotton thread to form loops.

### 4.4. Tension Measurements

The intact aortic rings and ventral prostate strips were mounted in a thermostated perfusion chamber maintained at 37 ± 0.5 °C, with a continuous flow of Tyrode’s solution (9 mL/min) that was consistently oxygenated with 100% O_2_. Changes in tension were isometrically measured with a force-displacement transducer (Harvard Apparatus, Holliston, MA, USA). The data were recorded using PowerLab (ADInstruments, Dunedin, New Zealand) and LabChart Pro v8.1.16 software (ADInstruments, Colorado Springs, CO, USA). Initially, aortic rings and prostate strips were stretched to a resting tension of 1.5 g and 1 g, respectively, and equilibrated under constant perfusion for 60 min. The perfusion was then replaced at least twice with high-KCl Tyrode’s solution (in mM: 40.4 NaCl, 100 KCl, 1.8 CaCl_2_, 0.9 MgCl_2_, 0.33 NaH_2_PO_4_, 10 glucose, and 10 HEPES, pH 7.4). The amplitude of KCl-induced contraction was considered as maximal contraction and used as the reference value for concentration-response curves elicited by phenylephrine.

### 4.5. Experimental Procedure

To record the effects of EO, 2AF, and 5MFF on muscle contraction, the aortic rings and prostate strips were initially pre-contracted with 0.3 µM or 1 µM phenylephrine, respectively. After initiating the contraction by phenylephrine, we allowed about 10 min for the contractions to stabilize and reach a consistent, steady-state level. Once this state was achieved, the tested compound—either EO, 2AF, or 5MFF—was cumulatively added in concentrations ranging from 0.01 µL/mL to 3 µL/mL. In experiments with the prostate, the maximal effect of EO was observed at a concentration of 1 µL/mL. The changes in tension at each concentration were expressed as relative values using 100% as the maximum contraction induced by phenylephrine.

The concentration-response curves for α_1_-adrenergic agonist phenylephrine in the prostate were generated through the cumulative introduction of phenylephrine, with concentrations ranging from 0.03 µM to 30 µM. The data obtained under perfusion with only Tyrode’s solution were considered as a control. To investigate the effects of tested compounds on α_1_-adrenergic receptors, the prostate strips were initially pretreated for 15 min with one of the compounds, i.e., EO, 2AF, or 5MFF, and then phenylephrine was added in an analogical cumulative pattern. The resulting concentration-response curves were compared with those obtained in the control. The changes in tension were represented as percentages, with 100% denoting the maximal contraction induced by Tyrode’s solution containing 100 mM KCl.

The effects of each tested compound were examined on separate preparations.

### 4.6. Evaluation and Statistical Analysis

Over the course of some experiments, a spontaneous decline in contraction was observed. This decline, or ‘rundown’, was eliminated in these experiments using nonlinear regression. To determine the IC_50_ (the half-maximal inhibitory concentration of EO, 2AF, and 5MFF), the EC_50_ (the half-maximal effective concentration of phenylephrine), and E_max_ (the maximal response induced by phenylephrine), the data were fitted to the Hill equation. A linear regression of the data was performed using the Schild equation to evaluate the antagonistic potency of EO. The slope and pA_2_ value that corresponded to the antagonist affinity were calculated [30].

Data are presented as the mean ± standard error of the mean (SEM). *n* refers to the number of experiments. The significance of differences was determined by one-way analysis of variance (ANOVA). *p* values less than 0.05 were considered statistically significant.

## 5. Conclusions

Our study shows that the essential oil extracted from *Elsholtzia ciliata* exhibits significant relaxing effects on smooth muscles in both the prostate and aorta. The inhibitory effect of EO on contraction is more pronounced in the prostate than in the aorta. The impact of EO might be attributed to its furan ring-containing dominant ketones. This study also introduced and confirmed the muscle-relaxing properties of two other furan ring compounds, 2-acetylfuran and 5-methylfurfural. Furthermore, EO potentially acts as a competitive antagonist for α1-adrenergic receptors within specific concentrations, suggesting that its muscle relaxant effect might be linked to the inhibition of α_1_-adrenoreceptors.

## Figures and Tables

**Table 1:** The half maximal inhibitory concentrations (IC_50_) of *E. ciliata* essential oil (EO), 2-acetylfuran (2AF), and 5-methylfurfural (5MFF) on phenylephrine-induced contractions of the rat prostate and aorta.

	IC_50_ (µL/mL)		
	EO	2AF	5MFF
Prostate, *n* = 5–10	0.24 ± 0.03 ^#^	0.65 ± 0.12 *	0.42 ± 0.09 *^,#^
Aorta, *n* = 3–5	0.72 ± 0.11	0.78 ± 0.22	0.96 ± 0.19

IC_50_ values were calculated from the curves presented in Figure 4. Data are presented as the mean ± SEM. * *p* < 0.05 vs. EO; ^#^ *p* < 0.05 vs. aorta, n indicates the number of prostate strips or aorta rings.

## Data Availability

Data is contained within the article.
